# Supplementary material for: Accurate MHC Motif Deconvolution of Immunopeptidomics Data Reveals a Significant Contribution of DRB3, 4 and 5 to the Total DR Immunopeptidome
Source: Front Immunol. 2022 Jan 26;13:835454. doi: 10.3389/fimmu.2022.835454 (PMC8826445; doi:10.3389/fimmu.2022.835454)
Supplement: Supplementary file 1 [file DataSheet_1.docx]

# Supplementary Material

| **Haplotype Group** | **IHW ID** | **Alternate ID** | **Class I HLA** | | | **Class II HLA (DR)** | | | |
| --- | --- | --- | --- | --- | --- | --- | --- | --- | --- |
|  |  |  | **A** | **B** | **C** | **DRB1** | **DRB3** | **DRB4** | **DRB5** |
| **DR52** | **IHW09023** | VAVY | 01:01 | 08:01 | 07:01 | 03:01 | 01:01 |  |  |
|  | **IHW09043** | BM21 | 01:01 | 41:01 | 17:01 | 11:01 | 02:02 |  |  |
|  | **IHW09060** | CB6B | 01:01 | 15:01 | 03:03 | 13:01 | 02:02 |  |  |
|  | **IHW09061** | 31227ABO | 02:01 | 18:01 | 07:01 | 14:01* | 02:02 |  |  |
|  | **IHW09063** | WT47 | 32:01 | 44:02 | 05:01 | 13:02 | 03:01 |  |  |
| **DR53** | **IHW09052** | DBB | 02:01 | 57:01 | 06:02 | 07:01 |  | 01:03N |  |
|  | **IHW09075** | DKB | 24:02 | 40:01 | 03:04 | 09:01 |  | 01:03 |  |
|  | **IHW09090** | AWELLS | 02:01 | 44:02 | 05:01 | 04:01 |  | 01:03 |  |
|  | **IHW09093** | BER | 02:01 | 13:02 | 06:02 | 07:01 |  | 01:03 |  |
| **DR51** | **IHW09013** | SCHU | 03:01 | 07:02 | 07:02 | 15:01 |  |  | 01:01 |
|  | **IHW09084** | CALOGERO | 02:01 | 40:02 | 02:02 | 16:01 |  |  | 02:02 |

**Supplementary Table 1.**

**HLA type of the Homozygous BLCLs including Class I (A, B, C) and Class II (DRB1, 3, 4 and 5)**

*: Not sequenced with exon3 for possible DRB1*14:54

**Supplementary Table 2.**

| **Haplotype Group** | **Cell Line**  **IHW ID** | **Primary DR** | **Count** | **Proportion** | **Secondary DR** | **Count** | **Proportion** |
| --- | --- | --- | --- | --- | --- | --- | --- |
| **DR52** | 9023 | DRB1*03:01 | 1545 | 61.6% | DRB3*01:01 | 964 | 38.4% |
|  | 9043 | DRB1*11:01 | 1164 | 79.7% | DRB3*02:02 | 297 | 20.3% |
|  | 9060 | DRB1*13:01 | 1285 | 54.4% | DRB3*02:02 | 1079 | 45.6% |
|  | 9061 | DRB1*14:01 | 1361 | 67.8% | DRB3*02:02 | 646 | 32.2% |
|  | 9063 | DRB1*13:02 | 964 | 43.8% | DRB3*03:01 | 1237 | 56.2% |
| **DR53** | 9052 | DRB1*07:01 | 2597 | 98% | DRB4*01:03N | 53 | 2% |
|  | 9075 | DRB1*09:01 | 1575 | 85.4% | DRB4*01:03 | 270 | 14.6% |
|  | 9090 | DRB1*04:01 | 2360 | 83.9% | DRB4*01:03 | 453 | 16.1% |
|  | 9093 | DRB1*07:01 | 1751 | 84.4% | DRB4*01:03 | 323 | 15.6% |
| **DR51** | 9013 | DRB1*15:01 | 1501 | 50.2% | DRB5*01:01 | 1490 | 49.8% |
|  | 9084 | DRB1*16:01 | 570 | 36.1% | DRB5*02:02 | 1010 | 63.9% |

**Peptide contribution of the primary and secondary HLA-DR molecules as obtained by MHCMotifDecon.**

**Supplementary Table 3.**

| **Haplotype Group** | **Cell Line**  **IHW ID** | **Overlap** | **Primary DR** | **Proportion** | **Secondary DR** | **Proportion** |
| --- | --- | --- | --- | --- | --- | --- |
| **DR52** | 9023 | 27.5% | DRB1*03:01 | 44.8% | DRB3*01:01 | 27.7% |
|  | 9043 | 1.5% | DRB1*11:01 | 73.1% | DRB3*02:02 | 25.4% |
|  | 9060 | 0.9% | DRB1*13:01 | 37.0% | DRB3*02:02 | 62.1% |
|  | 9061 | 3.5% | DRB1*14:01 | 59.3% | DRB3*02:02 | 37.2% |
|  | 9063 | 11.0% | DRB1*13:02 | 29.4% | DRB3*03:01 | 59.6% |
| **DR53** | 9052 | 0.8% | DRB1*07:01 | 98.4% | DRB4*01:03N | 0.83 |
|  | 9075 | 0.6% | DRB1*09:01 | 80.3% | DRB4*01:03 | 19.2% |
|  | 9090 | 7.0% | DRB1*04:01 | 75.8% | DRB4*01:03 | 17.2% |
|  | 9093 | 0.3% | DRB1*07:01 | 78.5% | DRB4*01:03 | 21.2% |
| **DR51** | 9013 | 1.7% | DRB1*15:01 | 51.3% | DRB5*01:01 | 47.0% |
|  | 9084 | 4.1% | DRB1*16:01 | 32.0% | DRB5*02:02 | 63.9% |

**The proportion of the DR ligandome predicted to be presented by either the primary (DRB1), secondary (DRB3, 4, or 5) or by both DR molecules.** HLA presentation was predicted using NetMHCIIpan-4.1 with a rank threshold of 1%.

**Supplementary Table 4.**

Immunopeptidome data generated for the study. Data cover 12-21mer peptides from the 11 Homozygous BLCLs described in supplementary table 1. The data are split into individual sheets, one for each cell line. Each sheet contains the peptides with associated source protein ID.


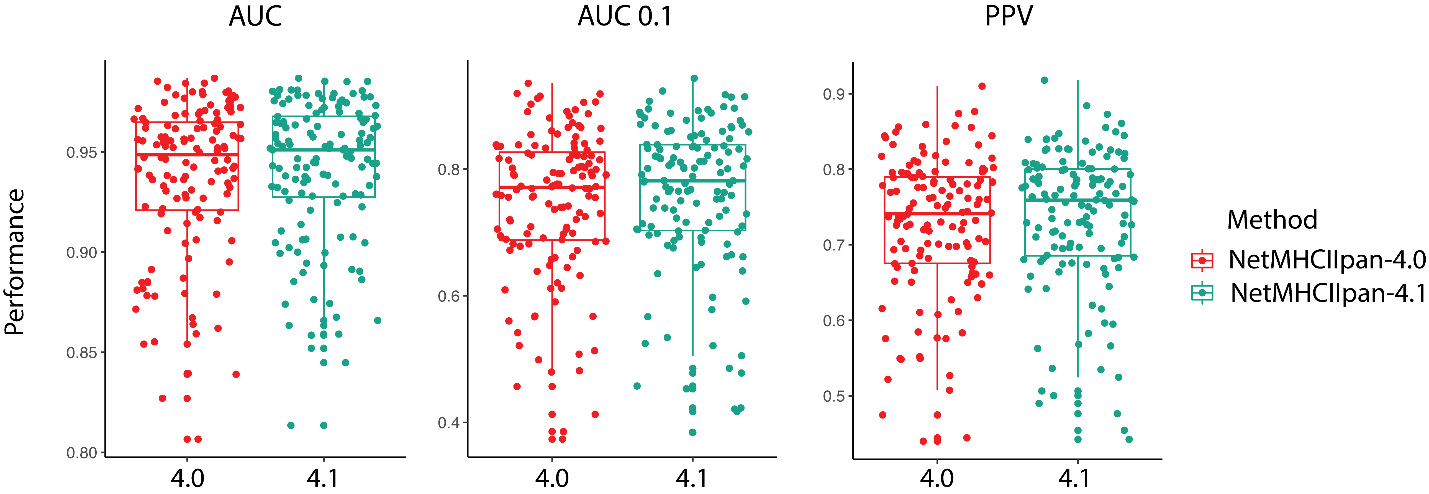


**Supplementary Figure 1.** Cross-validation performance evaluation on NetMHCIIpan 4.1 and NetMHCIIpan 4.0. Each dot in the plots refers to a single dataset. AUC 0.1 refers to the area under the ROC curve integrated up to a false positive rate of 10%, and PPV is the predicted positive values calculated from the proportion of positives with the top N highest predictions, where N is the total number of positives within the given dataset. NetMHCIIpan-4.1 significantly outperforms NetMHCIIpan-4.0 in all performance metrics (p<0.001, binomial test, in all cases).


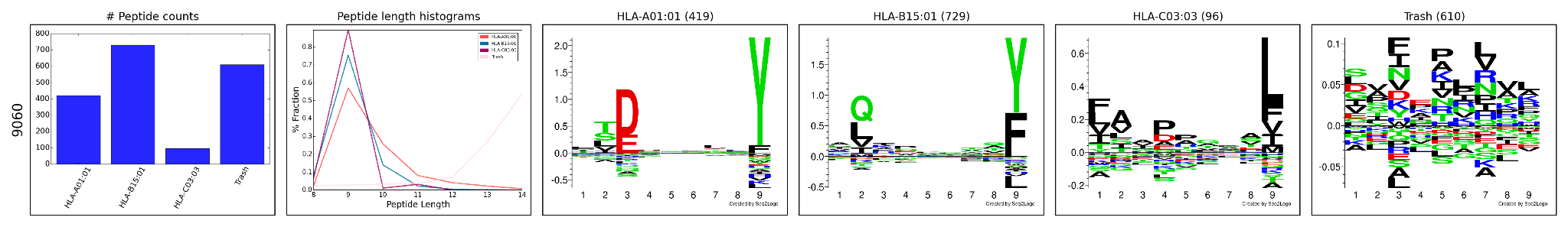


**Supplementary Figure 2.** MHCMotifDecon HLA class I motif deconvolution for the IHW09060 dataset using a trash bin threshold of 2%.


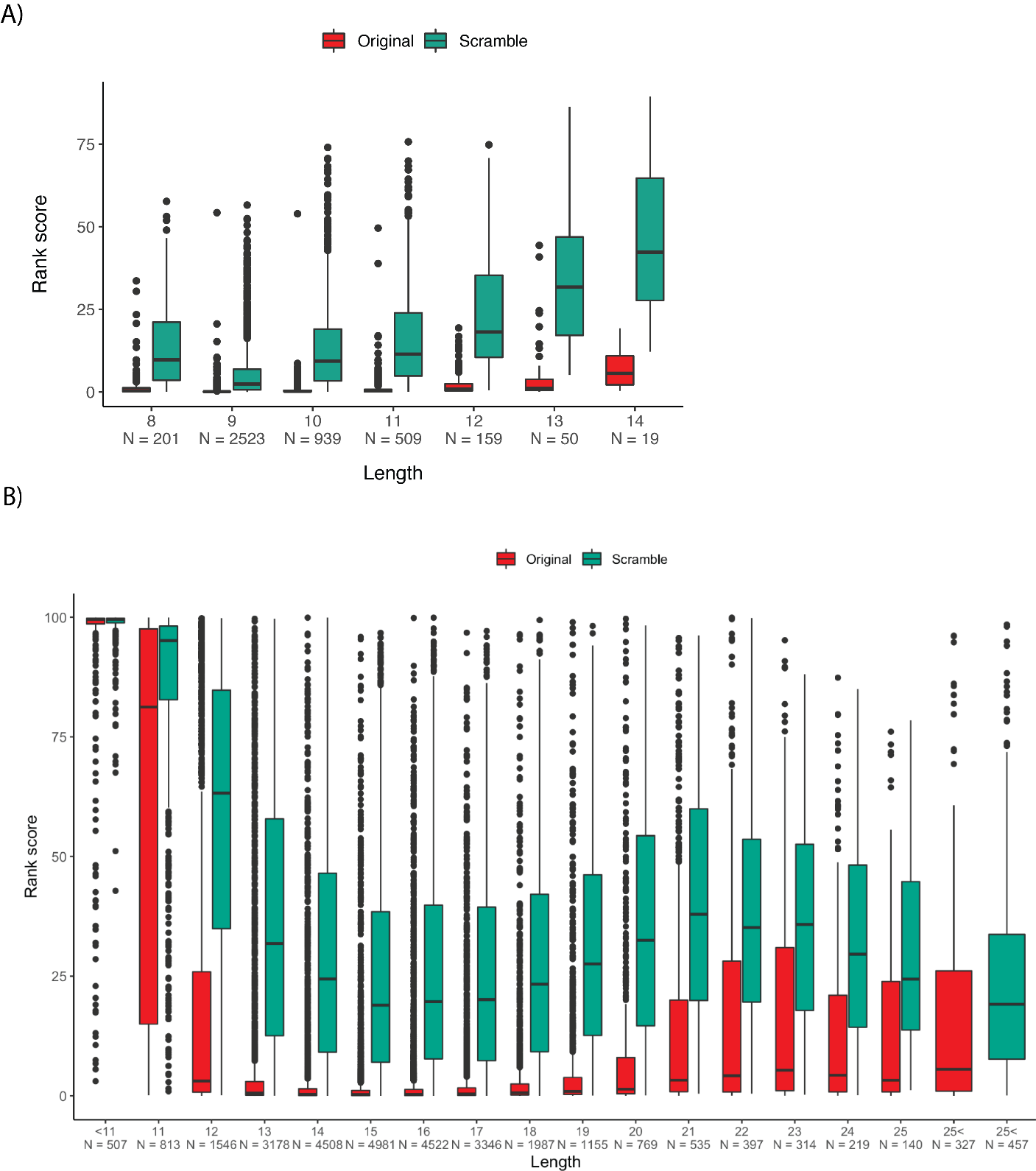


**Supplementary Figure 3. Comparison of rank scores per length after deconvolution of original and scrambled datasets with MHCMotifDecon**. In the scrambled dataset, the peptide sequences have the same amino acid composition but in a shuffled order (for details refer to methods section). 100 scrambled variations were generated for each peptide. For comparison a random sample of all the scrambled peptides matching the number of peptides in the original dataset were selected (for details on scrambled data refer to Methods section). To quantify the proportion of scrambled peptides that would be assigned to an HLA expressed by the cell line, the rank scores obtained by MHCMotifDecon were compared to those obtained from the original peptide data. **(A)** Shows the results for HLA class I, and **(B)** the results for HLA class II.
